# Burden of Acute Gastrointestinal Illness in Gálvez, Argentina, 2007

**DOI:** 10.3329/jhpn.v28i2.4885

**Published:** 2010-04

**Authors:** M. Kate Thomas, Enrique Perez, Shannon E. Majowicz, Richard Reid-Smith, Silvia Albil, Marcos Monteverde, Scott A. McEwen

**Affiliations:** ^1^ Department of Population Medicine, Ontario Veterinary College, University of Guelph, Guelph, Canada; ^2^ Pan American Health Organization, Rio de Janeiro, Brazil; ^3^ Centre for Food-borne, Environmental and Zoonotic Infectious Diseases, Public Health Agency of Canada, Guelph, Canada; ^4^ Laboratory for Foodborne Zoonoses, Public Health Agency of Canada, Guelph, Canada; ^5^ Centro de Desarrollo Agroalimentario, Municipality of Gálvez, Gálvez, Argentina; ^6^ Secretario Agencia Santafesina de Seguridad Alimentaria, Santa Fe, Argentina

**Keywords:** Cross-sectional studies, Developing country, Diarrhoea, Foodborne diseases, Morbidity, Population surveys, Recall bias, Waterborne diseases, Argentina

## Abstract

This study evaluated the magnitude and distribution of acute gastrointestinal illness (GI) in Gálvez, Argentina, and assessed the outcome of a seven-day versus 30-day recall period in survey methodology. A cross-sectional population survey, with either a seven-day or a 30-day retrospective recall period, was conducted through door-to-door visits to randomly-selected residents during the ‘high’ and the ‘low’ seasons of GI in the community. Comparisons were made between the annual incidence rates obtained using the seven-day and the 30-day recall period. Using the 30-day recall period, the mean annual incidence rates was 0.43 (low season of GI) and 0.49 (high season of GI) episodes per person-year. Using the seven-day recall period, the mean annual incidence rate was 0.76 (low season of GI) and 2.66 (high season of GI) episodes per person-year. This study highlights the significant burden of GI in a South American community and confirms the importance of seasonality when investigating GI in the population. The findings suggest that a longer recall period may underestimate the burden of GI in retrospective population surveys of GI.

## INTRODUCTION

Acute gastrointestinal illness (GI) causes significant morbidity, mortality, and socioeconomic burden worldwide (1, 2). Clean water, sanitation, and food safety are key components to preventing and controlling GI in the population (3). These public-health areas are at the forefront of the objectives and priorities of international public-health organizations and concerns of local public health workers (4–7). Understanding the magnitude, distribution, and demographic factors associated with GI is key for its mitigation (8). However, cases of GI tend to be under-reported by traditional surveillance techniques, which require cases to seek medical attention to be captured. To address this, numerous countries have conducted population-based studies to better estimate the burden of disease (8–19). With population-level baseline information, interventions, targeted surveillance, and research activities can be accurately evaluated. Likewise, the impacts of broader worldwide trends, such as globalization, climate change, and international travel and trade, on the magnitude and distribution of disease can be gauged. Additionally, within methodology of population-based studies, discussions on prospective and retrospective methods, selection of recall period, and recall bias are ongoing (18, 20, 21). Further research to evaluate these issues within the context of the burden of GI is needed.

In September 2006, the Ministry of Health of Argentina completed their first pilot study on the burden of GI in Diamante (Entre Rios province), which estimated a monthly GI prevalence of 8.2% (Rico O. Personal communication, 2006). Building from the pilot, we conducted a study in Gálvez (Santa Fe province) in 2007. The objectives of the Gálvez study were to determine the magnitude and distribution of GI in the population, describe its burden and clinical presentation, evaluate under-reporting, and identify the risk factors associated with GI. An additional objective was to assess the differences between a seven-day recall period and a 30-day recall period.

## MATERIALS AND METHODS

### Population baseline study

A cross-sectional, door-to-door survey of randomly-selected residents of Gálvez, Santa Fe, Argentina, was administered during 30 April 2007–21 May 2007 (Phase 1: high GI season) and 1–12 October 2007 (Phase 2: low GI season). Gálvez and the pilot location—Diamante—were conveniently selected by the Argentine Ministry of Health based on their suitability, willingness of local and regional authorities, feasibility of completing the studies, and availability of data based on local and regional surveillance activities. Gálvez has a population of approximately 18,500, is primarily an urban area surrounded by farmland and rural areas, and is divided into 15 neighbourhoods [Instituto Nacional de Estadistica y Censos. 2001 census data (www.indec.mecon.gov.ar) and 2000 Ciudad de Gálvez (www.unimedio.com/galvez)]. Designation of ‘high’ and ‘low’ seasons of GI was based on data contained in the municipal surveillance system housed at the Centro de Desarrollo de Agroalimentario (CeDA) Gálvez, Argentina. This surveillance system collects the monthly number of cases of GI in the community presenting at the local hospital and clinics.

Trained interviewers from the community conducted face-to-face interviews. Households were randomly selected proportionally by neighbourhood population from a community census using the Epidat software (version 3.1) (Pan American Health Organization, 2006). The individual in the household with the next birthday was selected to participate in the survey as is commonly done in population surveys to achieve a random sample (10, 14–17). If the selected individual declined or no one lived at the residence, the neighbouring house, that being the next closest house, was selected conveniently by the surveyor, as replacement. If the selected individual was aged less than 12 years, the parent or guardian answered the survey on their behalf. If the selected individual was aged 12–18 years, the parent, guardian, or child answered the survey at the discretion of the parent or guardian. All surveys were administered in Spanish.

### Sample size

Sample sizes were calculated using the Epi Info software (version 3.0) (Centers for Disease Control and Prevention, Atlanta, Georgia, USA, 2000), with a 2% allowable error and a 95% confidence level in a population of 18,500. In Phase 1 (high season of GI), the target sample sizes of 681 respondents (30-day recall period) and 725 respondents (7-day recall period) were based on expected monthly (8%) and weekly (2%) prevalence estimated from a prior pilot study in Diamante, Argentina. The prevalence estimated from Phase 1 were used as expected prevalence in Phase 2 (low season of GI), yielding the target sample size of 753 respondents for both 30- and seven-day recall periods. The total target sample size for the study was 2,912.

### Collection of data

The survey instrument (available upon request from the authors) was developed by modifying the survey tools used previously in Diamante, Argentina. Modifications to the Diamante pilot survey included revisions to some questions to improve their clarity and utility while additional questions pertaining to potential risk factors and recent antibiotic-use were incorporated. Respondents were asked if they had experienced any symptoms of diarrhoea in the previous seven or 30 days, depending on the survey recall period, where diarrhoea was defined as three or more loose stools in 24 hours. Individuals who suffered from chronic diarrhoea or diarrhoea caused by use of medications, laxatives, alcohol, or medical conditions, were considered non-cases. Additional questions asked about sociodemographic factors, secondary symptoms, number of missed school or work days, and whether hospitalization was required.

### Estimation of under-reporting

From the population survey, the percentage of cases who visited the local clinics and hospital was used for estimating the magnitude of under-reporting from the community level to the CeDA-managed municipal surveillance system, using the model shown in the burden of illness pyramid (Fig.).

**Fig.**
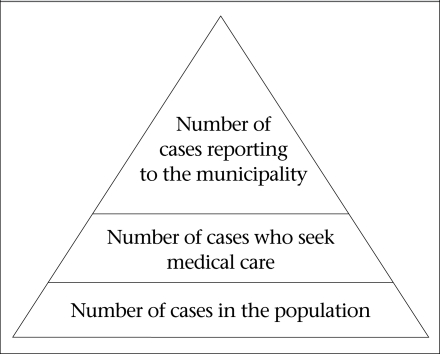
Theoretical burden of illness pyramid for Gálvez, Argentina, 2007

### Statistics

Data were manually entered into the Epi Info software (version 3.0) and managed using the Microsoft Access software. Analysis was performed using the SAS software (version 9.0) (SAS Institute Inc., Cary North Carolina, USA, 2004). Individuals responding ‘do not know’ or ‘unsure’ were excluded from the analysis of that question. Whether cases had used antibiotics in the four weeks before illness was compared with whether non-cases had used antibiotics in the four weeks before interview to assess the effect of recent antibiotic-use.

Univariable analysis was performed on the overall dataset (both recall periods and study phases). The null hypothesis of no association between the presence of GI and the individual potential risk factors was tested using the Fisher's Exact test or the Monte Carlo estimation of the Fisher's Exact test in the SAS software. A weighted multivariate logistic regression model was built manually beginning with those variables that had a p value of <0.25 in Fisher's Exact test in univariate analysis (22). Weighting was used for correcting for differences in neighbourhood sampling fractions. All remaining variables were offered to the model; however, only variables with a p value of <0.05 (Wald's test) were kept in the final model. The differences between medians were tested using the median test in the SAS software.

The primary outcome measures of monthly and weekly prevalence were defined as the number of respondents reporting GI in the previous 30 or seven days respectively, divided by the total number of respondents for the 30- or seven-day surveys. The prevalence, incidence rate, and incidence proportion calculations were also performed (23); the formulae are shown in the Appendix.

Using the burden of illness model shown in the figure, the estimate of under-reporting was generated via stochastic modelling in @RISK (student version) (Palisade Corporation, Ithaca, New York, USA) as an add-on to Microsoft Excel. The Beta form (*a, b*) where *a*=number of cases who seek medical care +1 and *b*=number of cases-number of cases who seek medical care + 1, was used for estimating under-reporting between the bottom and the middle step of the pyramid (% of cases who seek care) (24). The percentage of cases reported to the municipal surveillance system was assumed to be 100%; therefore, the inverse of the percentage of cases who seek care was considered to be the estimated under-reporting fraction.

To facilitate international comparisons, Majowicz *et al*. proposed a minimum set of reported results and a standard symptom-based case definition for GI of three or more loose stools or any vomiting in 24 hours, excluding those (a) with cancer of the bowel, irritable bowel syndrome, Crohn's disease, ulcerative colitis, cystic fibrosis, coeliac disease, or any other chronic illness with symptoms of diarrhoea or vomiting, or (b) who report that their symptoms were due to drugs, alcohol, or pregnancy (25). Although the definition of our study did not capture ‘vomiting only’ cases, we still report the suggested minimum set of results, using the study definition to facilitate international study comparisons.

### Ethics

The Human Subjects Committee of the University of Guelph Research Ethics Board (Guelph, Ontario, Canada), in partnership with the Ministry of Health of Argentina, approved the study. Signed, informed consent was obtained from all participants or the parent/guardian if the participant was a minor.

## RESULTS

### Magnitude, distribution, and burden

The demographic distribution of Gálvez residents versus survey respondents are shown in Table 1, along with the prevalence, annual incidence rate, annual incidence proportion, and prevalence by demographic characteristics. The overall annual incidence rate varied between 0.46 and 1.68 episodes per person-year, for the 30-day and seven-day recall periods respectively. Statistically significant higher annual incidence proportions were observed in Phase 1 (high season of GI) compared to Phase 2 (low season of GI) for the seven-day recall period.

**Table 1.** Respondent representativeness, demographic distribution, and prevalence of acute gastrointestinal illness per study phase and recall period in Gálvez Argentina, 2007

Variable and measures of disease	Gálvez residents* (n=18,542)	Phase 1: 30-day		Phase 1: 7-day		Phase 2: 30-day		Phase 2: 7-day	
		Survey respondents (n=680)	Prevalence (n=27)	Survey respondents (n=724)	Prevalence (n=36)	Survey respondents (n=755)	Prevalence (n=26)	Survey respondents (n=756)	Prevalence (n=11)
Demographic variables (%)									
Gender									
Male	48.3	38.2	5.4	40.7	3.9	34.0	1.4	36.8	4.3
Female	51.7	61.8	4.7	59.3	4.1	66.0	1.5	63.2	3.0
Age (years)									
0–4	7.2	1.7	14.3	2.0	9.1	2.1	0.0	1.1	6.3
5–9	7.7	1.1	6.7	2.1	28.6	1.7	0.0	1.7	7.7
10–19	16.9	5.1	0.0	7.3	14.7	10.5	1.5	9.0	10.1
20–59	49.1	69.0	3.7	65.2	3.3	52.7	1.6	58.6	2.3
60+	19.1	23.2	9.0	23.5	2.6	32.9	1.3	29.6	2.8
Education									
<6 years old (not yet in school)	NA	3.9	14.3	2.0	15.4	1.5	0.0	3.6	11.5
Illiterate	NA	1.1	12.5	1.5	0.0	1.5	0.0	1.1	25.0
Primary	NA	43.4	6.1	43.2	3.9	49.5	1.4	48.4	2.8
Secondary	NA	37.2	3.5	46.1	4.7	37.2	1.4	36.6	3.4
Tertiary	NA	9.1	1.5	7.8	0.0	7.3	3.7	9.3	2.9
University	NA	3.2	4.4	3.2	0.0	2.0	0.0	2.5	0.0
Number of people in household									
1–4	NA	79.1	5.0	76.2	3.5	76.0	1.2	80.5	3.0
5+	NA	20.9	4.7	23.8	5.6	24.0	2.2	19.5	5.4
Magnitude									
Prevalence (95% CI)‡	NA	3.97% (2.5–5.4)	4.97% (3.4–6.6)	3.44% (2.3–5.0)	1.46% (0.7–2.6)
Incidence rate¶ (95% CI)	NA	0.49 (0.31–0.68)	2.66 (1.83–3.58)	0.43 (0.28–0.63)	0.76 (0.35–1.40)
Incidence proportion§ (95% CI)	NA	38.9% (26.5–49.1)	93.0% (83.5–97.2)	34.7% (24.2–46.7)	53.4% (29.2–75.3)

*2001 census data, Instituto Nacional de Estadistica y Censos (www.indec.mecon.gov.ar);

‡Weekly or monthly prevalence according to seven-day or 30-day recall period;

¶Annual incidence rate per person-year;

§Annual incidence proportion;

CI=Confidence interval;

NA=Not available/applicable

The proportion of the study population who were female or aged over 19 years was larger than the target population of Gálvez. The median age of cases (46.5 years) and non-cases (46.6 years) for the full dataset was not statistically different (p=0.92). The response rate of 61.1% for Phase 2 was calculated by dividing the number of completed surveys by the number of households visited. Denominator data were not available for Phase 1, and the response rate was, thus, not calculated.

Table 2 shows the type and frequency of secondary symptoms. Headache and muscle-pain were most often reported, followed by vomiting and fever. Bloody diarrhoea was only reported by cases in the Phase 1, in the 30-day recall period.

**Table 2.** Symptoms and their duration for both study phases and recall periods combined, Gálvez, Argentina, 2007

Secondary symptom	No. of cases reporting secondary symptoms (n=100)
Headache	23
Fever	10
Muscle-pain	23
Nausea	4
Vomiting	6
Cramps	4
Stomach pain	1
Bloody diarrhoea	4
Duration (days)	
Range	0.5–28
Median	3
Mean	3.4

The overall number of missed work and school days of cases and of caretakers is shown in Table 3. In Phase 1, a greater proportion of cases missed work or school, and with a higher maximum number of days missed, compared to Phase 2. However, in Phase 2, a larger proportion of cases had family members who missed work or school to take care of them.

**Table 3.** Days of missed work and school by cases and caregivers for both study phases and recall periods combined, Gálvez, Argentina, 2007

Variable	No. of cases (n=100)
Number of cases who missed work due to illness	19
Median number and range of missed days	2 (1–8)
Number of cases who missed school due to illness	10
Median number and range of missed days	2.5 (1–7)
Number of cases with caregivers who missed work or school days	7
Median number and range of caregivers’ missed days	1 (1–3)

### Univariate and multivariate analyses

In the overall dataset (n=2,915), study phase (p<0.05), age (p<0.05), neighbourhood of residence (p<0.05), level of education (p=0.08), occupation (p=0.25), number of people in the household (p=0.19), ownership of a rabbit (p=0.08), ownership of a dog (p=0.22), and ownership of a chicken (p=0.14) were associated at the preliminary univariate level (p<0.25) with being a case of GI. Variables not associated were sex (p=0.46), number of bedrooms in the household (p=0.82), use of antibiotics in the four weeks before illness (p=1.00), ownership of a cat (p=0.26), a bird (p=0.87), a cow (p=1.00), a horse (p=1.00), a sheep (p=1.00), a goat (p=1.00), a turtle (p=1.00), a fish (p=1.00), and ownership of any pet (p=0.32). A final multivariate model included significant predictor variables (p<0.05) of study phase, age, and neighbourhood of residence (Table 4).

**Table 4.** Final multivariate model of risk factors associated with acute gastrointestinal illness in Gálvez, Argentina, 2007

Variable	Frequency	Odds ratio (95% CI)	p value
Study phase			0.0003
Phase 1 (High)	1,376	2.14 (1.41–3.24)	
Phase 2 (Low)	1,504	Referent	
Age (years)			0.0009
0–4	49	3.25 (1.15–9.17)	
5–9	48	2.87 (0.89–9.30)	
10–19	233	3.24 (1.75–6.02)	
20–59	1,759	Referent	
60+	791	1.65 (1.04–2.16)	
Neighbourhood*			0.0445
A	38	0.53 (0.00–74.44)	
B	164	0.94 (0.32–2.80)	
C	235	0.25 (0.08–0.82)	
D	435	Referent	
E	261	0.14 (0.03–0.54)	
F	160	0.59 (0.19–1.81)	
G	172	0.87 (0.33–2.29)	
H	87	0.63 (0.09–4.58)	
I	212	0.53 (0.21–1.37)	
J	196	0.70 (0.28–1.73)	
K	267	0.35 (0.14–0.85)	
L	175	0.56 (0.19–1.65)	
M	113	0.17 (0.01–2.96)	
N	295	1.24 (0.72–2.14)	
O	65	1.44 (0.22–9.26)	

*Neighbourhoods have been given an identifying letter to maintain confidentiality;

CI=Confidence interval

### Use of medical system

Medications used by cases to treat symptoms, medical facilities visited by cases, and reasons for not seeking medical care are reported in Table 5. Antidiarrhoeals and analgesics were used most frequently, followed by antibiotics with and without pres-cription. Of those cases who sought medical care, private clinics and the public hospital were most frequently visited. In total, two cases required hospitalization for their illness for two days and eight days respectively, both during Phase 1. ‘Self-medication’ and ‘not thinking the illness was important enough to seek medical care’ were the most common reasons for not seeking medical attention.

**Table 5.** Medications and access to medical care, for both study phases and recall periods combined, Gálvez, Argentina, 2007

Variable	No. of cases (n=100)
Medications to treat symptoms	
Analgesics	11
Antibiotics (with and without prescription)	7
Antidiarrhoeals	16
Antiinflammatories	2
Diet	1
Sought medical care	
Yes	26
No	74
Location of medical care sought*	
Private clinics	16
Public clinics	1
Public hospitals	7
Unsure/did not respond	3
Reasons for not seeking medical care	(n=74)
Self-medication	9
Natural remedies	5
Did not have time	1
Did not think it was important	20
Unsure/did not respond	39

*Some cases visited more than one location; so, the total may exceed 100%

### Estimation of under-reporting

Table 6 shows the mean, minimum and maximum percentages of cases who sought medical care. Assuming that all cases who sought medical care are reported to the surveillance system, the average number of cases of GI in the community for each case in the surveillance system ranged from 2.6 (minimum=1.5, maximum=7.4) to 4.3 (minimum=1.7, maximum=90.1), depending on the study phase and the recall period.

**Table 6.** Number and mean, minimum and maximum percentages of cases who sought medical attention and estimated under-reporting, for both study phases and recall periods, Gálvez, Argentina, 2007

Pyramid step	Phase 1				Phase 2			
	30-day mean (n=680) (min, max)		7-day mean (n=724) (min, max)		30-day mean (n=680) (min, max)		7-day mean (n=724) (min, max)	
Total surveyed	680		724		755		756	
Cases	27		36		26		11	
Visited MD	10	38.4	8	23.6	6	24.9	2	23.1
	(13.6, 65.2)		(6.0, 51.9)		(4.4, 52.0)		(1.1, 60.2)
Under-reporting factor* (95% CI)		2.6		4.2		4.0		4.3
	(1.5–7.4)		(1.9–16.7)		(1.9–22.7)		(1.7–90.1)

*Estimated number of cases in the population per case reported to the municipal surveillance system;

CI=Confidence interval;

MD=Medical doctor/physician;

Min=Minimum;

Max=Maximum

### Comparison of standard case definition

Table 7 reports the proposed minimum set of results of this study, thus allowing for international comparisons. Using a subset of the proposed standard case definition, no statistically significant differences were observed between the incidence of GI in males and females within a given recall period nor in the percentage of cases with symptoms on the day of interview between the study phases and the recall periods.

**Table 7.** Minimum set of results proposed for studies of acute gastrointestinal illness (25) for both study phases and recall periods, Gálvez, Argentina, 2007*

Categories of minimum set of results	Phase 1		Phase 2	
	30-day	7-day	30-day	7-day
Annual incidence per person-year (95% CI)	0.49	2.66	0.43	0.76
	(0.31–0.68)	(1.83–3.58)	(0.28–0.63)	(0.35–1.40)
Annual incidence per person-year in males	0.48	2.92	0.53	0.76
Annual incidence per person-year in females	0.50	2.50	0.37	0.77
Mean age (years) of cases	37	52	39	46
Mean duration (days) of illness	4.4	2.4	3.0	5.9
Cases with bloody diarrhoea (%)	15	0	0	0
Cases who saw a physician (%)	37	22	23	18
Cases submitting a stool sample for testing (%)	11	6	0	0
Cases with respiratory symptoms (%)	(…)†	(…)†	(…)†	(…)†
Cases with symptoms still ongoing at time of interview (%)	15	14	15	18

*Study definition for case of GI was anyone who had experienced 3 or more loose stools in 24 hours

†Data not collected. Survey respondents were not asked about respiratory symptoms;

CI=Confidence interval;

GI=Gastrointestinal illness

## DISCUSSION

This study provides the first population-based estimates of the magnitude, distribution, and burden of GI in an Argentinean community. The study also provided an opportunity to evaluate the effect of the retrospective recall period (seven-day vs 30-day recall) on estimates generated from a GI survey.

In both the phases of the study, the seven-day recall period yielded higher annual estimates of GI than the 30-day recall period. Assuming that recall bias is minimized if the recall period is shorter, this is contrary to the suggestion that ‘telescoping’ past illnesses into the observation period causes overestimates of disease in the population when using retrospective methods as suggested by Wheeler *et al*. (18). These results may be evidence of a recall-bias effect in the opposite direction such that the true burden of disease is actually under-estimated when a longer recall period is used. This may be due to forgetting episodes of ‘familiar illnesses’, such as GI, or more easily remembering illnesses that are perceived as severe (26). Further research on the mechanisms of this potential bias is warranted.

We found that age, study phase, and neighbourhood of residence were all significantly associated with GI. The odds of GI were 2.14 times higher in the ‘high’ season (phase 1) compared to the ‘low’ season (Phase 2). The odds of GI were the greatest among the young (those aged less than 20 years) and the elderly (those aged over 59 years) when compared with the referent group (aged 20–59 years), which is similar to other reported studies (9, 12, 14, 16, 17, 19). Three neighbourhoods had significantly lower odds of GI compared to the referent neighbourhood. These three neighbourhoods are located on the northwest, east, and southeast borders of the referent neighbourhood. Sociodemographic information is not available at the neighbourhood level.

Municipal surveillance data for Gálvez support the seasonal trend observed in this study; during the same timeframe, surveillance data showed a peak of GI prevalence in the high season (Phase 1) that was approximately three times the prevalence seen in the low season (Phase 2). A seasonal effect was also observed in a Cuban study in 2005–2006, where the prevalence of GI was approximately 2–5 fold greater in the rainy season compared to the dry season (10). Likewise, in Gálvez, the high season of GI coincided with more rainfall, and the low season of GI coincided with less rainfall [Oliveros Weather Station, Santa Fe, Argentina, Instituto Nacional de Tecnología Agropecuaria. 2005–2008 meteorological data (www.inta.gov.ar)]. Interestingly, the significantly higher odds associated with the ‘high’ season in this study was more pronounced for the seven-day vs the 30-day recall period. This phenomenon warrants more investigation.

Gender was not significantly associated at the univariable level with GI in any recall periods or study phases. However, it was striking that, in Phase 1 (but not Phase 2), all cases aged less than15 years were male (n=8, data not shown). Similarly, results of a Cuban study indicate that, when controlling for season, sentinel site, and age-group, there was a higher risk for males than for females, supporting this potential relationship (10). A study in England and Wales on demographic determinants of *Campylobacter*-associated infections also found an increased risk among males between birth and 17 years of age (27). The potential higher risk of GI of young males in the high season should be pursued in further research on behavioural and other risk factors.

Our results indicate that there are more cases in the community than are captured by the local GI surveillance systems, demonstrating that the true burden of GI is larger than typically detected by surveillance. Similar under-reporting has been found by several other studies in developed countries (9, 12, 14, 15, 17–19, 28). We assumed that all cases who sought medical care were captured by the municipal surveillance system but could not verify this. Any human error in reporting of cases or misclassification of cases at the hospital or clinic level would contribute to further under-estimation of the true burden.

The strict case definition used here was selected to be consistent with the previous pilot study in Argentina and was specifically chosen to reduce potential misclassifications of cases of non-infectious causes of GI symptoms (e.g. alcohol consumption). However, some infectious GI cases with vomiting as the sole symptom or less than three episodes of diarrhoea in 24 hours may have been excluded using this definition, and if so, this would cause some under-estimation of the true burden in the community.

Our findings are similar to those of others who have applied the proposed symptom-based case definition (25), with the exception of the incidence calculations for the Phase 1, in the seven-day recall period. However, our results are based on two time periods selected to represent the ‘high season of GI’ and the ‘low season of GI’ in the community and cannot, thus, be applied directly as the full annual estimates.

In Phase 1 of the study, we observed more cases in the seven-day recall period than in the 30-day recall period. This is surprising given that these two survey recall periods occurred during the same calendar time period. Further investigation of this is necessary, potentially examining multiple recall periods, study locations, and times.

A potential limitation of the present study was the retrospective methodology used. Retrospective methods may have more recall bias and, thus, under ideal conditions, prospective methodology is preferred (18). This is somewhat compensated by the advantage that we used similar methods in numerous other retrospective studies, thereby enabling comparison with these studies.

Another limitation of the study may be selection bias as the age and gender distributions of the study participants differed from those of the reference community. Additionally, lack of denominator data for Phase 1 prevented calculation of the response rate. However, since the structure and management of both the study phases were identical, it is likely that there is not a large difference between response rates of the two phases. Moreover, a response rate of 61% was achieved for Phase 2 of the study, which is on the high-end of the range of response rates from other published retrospective surveys (25). The door-to-door methodology likely contributed to the relatively high response rate. Provided that there are no differences between the responders and the non-responders in terms of confounding characteristics and the risk of GI, non-response should not impact our results.

Those in institutions and hospitals were not included as part of the study population. It is, thus, possible that cases of GI who resided in these locations were missed and may cause an under-estimation of the true burden.

This study builds on the pilot burden of GI research conducted by the Argentina Ministry of Health and is the first publication of this kind from Argentina. It contributes to the growing understanding of GI in the population and highlights the significant burden of GI in this Argentine community. It presents evidence suggesting that a shorter recall period may be more valid for retrospective population surveys of GI. It demonstrates associations between GI and age, neighbourhood of residence, and season. It provides the proposed required results for international comparison using a subset of the proposed standard case of GI definition.

## ACKNOWLEDGEMENTS

The Public Health Agency of Canada, the Pan American Health Organization, the Centro de Desarrollo Agroalimentario de Galvez (CeDA), and the University of Guelph provided financial support.

The authors acknowledge the support of the CeDA, Argentina, the cooperation of the residents of Gálvez, Argentina, the support of the ministries of Health (Santa Fe province and Argentina), the cooperation of the health facilities and laboratory technicians serving the residents of Gálvez, Argentina, Fernando Olea for assistance in data entry, Aamir Fazil for technical assistance with @Risk modelling, and William Sears for technical assistance with the SAS software.

## APPENDIX

Formulae for calculating prevalence, incidence rate, and incidence proportion (23)

**Table:** Values

Variable	Phase 1		Phase 2	
	30-day	7-day	30-day	7-day
No. of cases	27	36	26	11
Total no. at risk	680	724	755	756
No. of withdrawals	0	0	0	0
No. of days of the recall period	30	7	30	7
